# Plasma Mediated Chlorhexidine Immobilization onto Polylactic Acid Surface via Carbodiimide Chemistry: Antibacterial and Cytocompatibility Assessment

**DOI:** 10.3390/polym13081201

**Published:** 2021-04-08

**Authors:** Kadir Ozaltin, Antonio Di Martino, Zdenka Capakova, Marian Lehocky, Petr Humpolicek, Tomas Saha, Daniela Vesela, Miran Mozetic, Petr Saha

**Affiliations:** 1 Center of Polymer Systems, Tomas Bata University in Zlin, Trida Tomase Bati 5678, 760 01 Zlin, Czech Republic; dimartino@tpu.ru (A.D.M.); capakova@utb.cz (Z.C.); lehocky@utb.cz (M.L.); humpolicek@utb.cz (P.H.); dvesela@utb.cz (D.V.); saha@utb.cz (P.S.); 2 Research School of Chemistry and Applied Biomedical Sciences, Tomsk Polytechnic University, Lenin Av. 30, 634050 Tomsk, Russia; 3 Faculty of Technology, Tomas Bata University in Zlin, Vavreckova 275, 760 01 Zlin, Czech Republic; 4 Footwear Research Center, University Institute, Tomas Bata University in Zlin, Nad Ovcirnou 3685, 760 01 Zlin, Czech Republic; tsaha@utb.cz; 5 Department of Surface Engineering and Optoelectronics, Jozef Stefan Institute, Jamova Cesta 39, 1000 Ljubljana, Slovenia; miran.mozetic@ijs.si

**Keywords:** chlorhexidine, polylactic acid, biomaterial associated infection, plasma treatment, cytocompatibility

## Abstract

The development of antibacterial materials has great importance in avoiding bacterial contamination and the risk of infection for implantable biomaterials. An antibacterial thin film coating on the surface via chemical bonding is a promising technique to keep native bulk material properties unchanged. However, most of the polymeric materials are chemically inert and highly hydrophobic, which makes chemical agent coating challenging Herein, immobilization of chlorhexidine, a broad-spectrum bactericidal cationic compound, onto the polylactic acid surface was performed in a multistep physicochemical method. Direct current plasma was used for surface functionalization, followed by carbodiimide chemistry to link the coupling reagents of N-(3-Dimethylaminopropyl)-N′-ethylcarbodiimide hydrochloride (EDAC) and N-Hydroxysuccinimide (NHs) to create a free bonding site to anchor the chlorhexidine. Surface characterizations were performed by water contact angle test, X-ray photoelectron spectroscopy (XPS) and scanning electron microscope (SEM). X-ray photoelectron spectroscopy (XPS) and scanning electron microscope (SEM). The antibacterial activity was tested using *Staphylococcus aureus* and *Escherichia coli*. Finally, in vitro cytocompatibility of the samples was studied using primary mouse embryonic fibroblast cells. It was found that all samples were cytocompatible and the best antibacterial performance observed was the Chlorhexidine immobilized sample after NHs activation.

## 1. Introduction

Biodegradable polymers, produced from renewable sources, are alternatives to conventional synthetic polymers with their competitive mechanical properties, biocompatibility, processability, thermal stability, low-cost and environmentally-friendly properties [1,2,3]. Polylactic acid (PLA) is one of the most widely used biodegradable polymers in biomedical applications, such as surgical plates, suture yards, and screws [2,3]. Like every other biomaterial used in living tissue as an implant, PLA surface is also open to endogenous or exogenous bacterial contamination. Such contamination may cause nosocomial infection during hospitalization, followed by patient discomfort, extended hospitalization time, external drug load to recover, post-operation to remove the implant, and even morbidity [3,4,5,6,7,8,9,10,11]. Biofilm formation on a biomaterial surface is a multistep process, which begins by bacterial contamination, mediated by physicochemical interaction on the surface, followed by bacterial adhesion through hydrogen bonds and proliferation by multilayering and clustering. Composed bacterial strain secretes an extracellular matrix (consist of polysaccharides, nucleic acids and proteins) to cover the colonies and creates a biofilm [11,12]. The biofilm ruptures after reaching the critical amount of bacteria and releases to the surrounding tissue, resulting in potentially serious infections. Removing the existing biofilm is challenging by drug treatment and in most cases, the solution is the removal of the implant. Therefore, bacterial contamination needs to be inhibited at the first stage of adhesion. Such bacterial adhesion and biofilm formation depend on surface charge and density, the chemical composition of the surface, its topology (roughness) and hydrophilicity [4,13]. Since only the biomaterial’s surface is in contact with the living tissue and environment during the implantation, creating an antibacterial surface to prevent bacterial adhesion is a valid and convenient approach, instead of blending the bulk material with antibacterial agents. In this way, antibacterial drug loading can be lowered to avoid the patient from the side effects of antibiotics as well as reduce the material cost and its release to the human body is controlled by covalent immobilization. One of the most commonly used ways to fabricate an antibacterial surface to prevent bacterial adhesion is using broad-spectrum antibacterial agents to immobilize on the biomaterials surface. Chlorhexidine (CHx) is a broad-spectrum bactericidal cationic compound belonging to the biguanide family and is toxic to both Gram-negative and Gram-positive bacteria [14,15,16,17,18]. It is widely used as an ingredient in household disinfectants, skin/hand antisepsis, hospital disinfectants, dental cleaning products, and cosmetics [17,19,20]. The action mechanism of the CHx targets bacterial cell membrane damage by electrostatic interactions of cationic CHx with anionic groups in the bacterial lipid layer to reduce cell viability or even finalize by cell death [19,20,21].

Immobilization of the CHx onto an inert surface such as the poly(lactic) acid (PLA) surface is challenging due to its high hydrophilic nature and lack of free bonding groups. Antibacterial surface coating by the plasma mediated multistep physicochemical method is a promising technique to overcome this drawback. Plasma treatment is a non-thermal, fast and effective process without using any chemicals or toxins. Since most of the polymer surfaces lack active functional groups for further chemical bonding and they are mostly hydrophilic, plasma treatment can be used to functionalize the polymer surface by plasma particles to create oxygen-containing functional groups (such as hydroxyl, carbonyl, carboxyl) and also increase the surface wettability/hydrophilicity by plasma etching [22,23,24,25,26,27,28,29,30,31]. The bulk material properties are not influenced by plasma treatment therefore its interaction is limited to the nanoscale [32,33]. In addition to antibacterial activity, the cytotoxicity behavior of the antibacterial containing biomaterials is crucial because most of the antibacterial agents are toxic to cells, therefore their usage needs to be moderated to allow cell growth on the biomaterials after implantation.

In this work, a plasma mediated multistep physicochemical method was used to immobilize CHx onto the PLA surface. Apart from plasma functionalization, carbodiimide chemistry was applied using the coupling reagents of *N*-(3-Dimethylaminopropyl)-*N*′-ethylcarbodiimide hydrochloride (EDAC), and *N*-Hydroxysuccinimide (NHs) to increase the anchoring of CHx onto the surface. Surface hydrophilicity analysis was carried out using a water contact angle test, chemical analysis to observe elemental changes was performed by x-ray photoelectron spectroscopy, and surface morphology was investigated with a scanning electron microscope. Antibacterial performance of the samples was tested against *Staphylococcus aureus* (CCM 4516) as Gram-positive and *Escherichia coli* (CCM 4517) as Gram-negative representatives. Finally, in vitro cytocompatibility of the samples was studied using primary mouse embryonic fibroblast cells.

## 2. Materials and Methods

### 2.1. Materials

**Figure 1:**
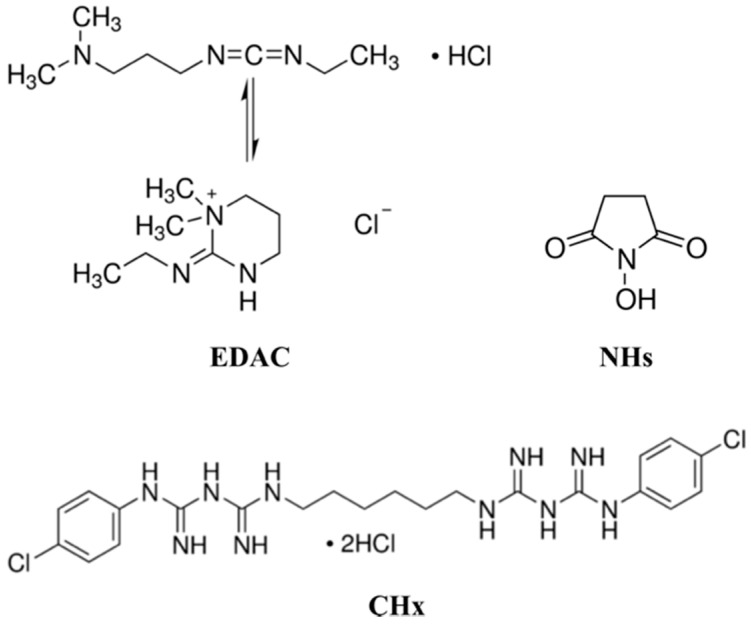
Chemical structures of N-(3-Dimethylaminopropyl)-N’-ethylcarbodiimide hydrochloride (EDAC), N-Hydroxysuccinimide (NHs) and Chlorhexidine dihydrochloride (CHx).

The pellet form of poly(lactic acid) (PLA) 4032 D was obtained from Nature Works (Blair, NE) to use as a substrate. The reagents of *N*-(3-Dimethylaminopropyl)-*N*′-ethylcarbodiimide hydrochloride (EDAC), *N*-Hydroxysuccinimide (NHs) and Chlorhexidine dihydrochloride (CHx) were purchased from Sigma-Aldrich and their chemical compositions are given in Figure 1. PLA pellets were first dried in an oven at 60 C overnight, then pressed into the shape of sheets in 150 µm thickness by compression molding at 180 C. PLA sheets were then cut into the square form of 50 × 50 mm, gently washed with distilled water, and subsequently dried at room temperature; hereafter referred to as PLA. The solutions of 0.1% (*w*/*v*) EDAC and NHs were prepared in distilled water and CHx in 70% (*v*/*v*) isopropanol.

### 2.2. Surface Modification

A 40 kHz direct current (DC) plasma reactor (Diener-PICO, Ebhausen, Germany) with a volume of 3 dm^3^ was used to functionalize the PLA surfaces before chemical agent immobilization. Ambient air was used as a discharge gas with 20 standard cubic centimeters per minute (sccm) flow rate and the reactor power was set to 50 watts. Each side of the PLA samples was subjected to DC plasma for 60 s under 50 Pa vacuum chamber pressure; hereafter referred to as PLA_DC.

### 2.3. Immobilization of the Chlorhexidine

**Figure 2:**
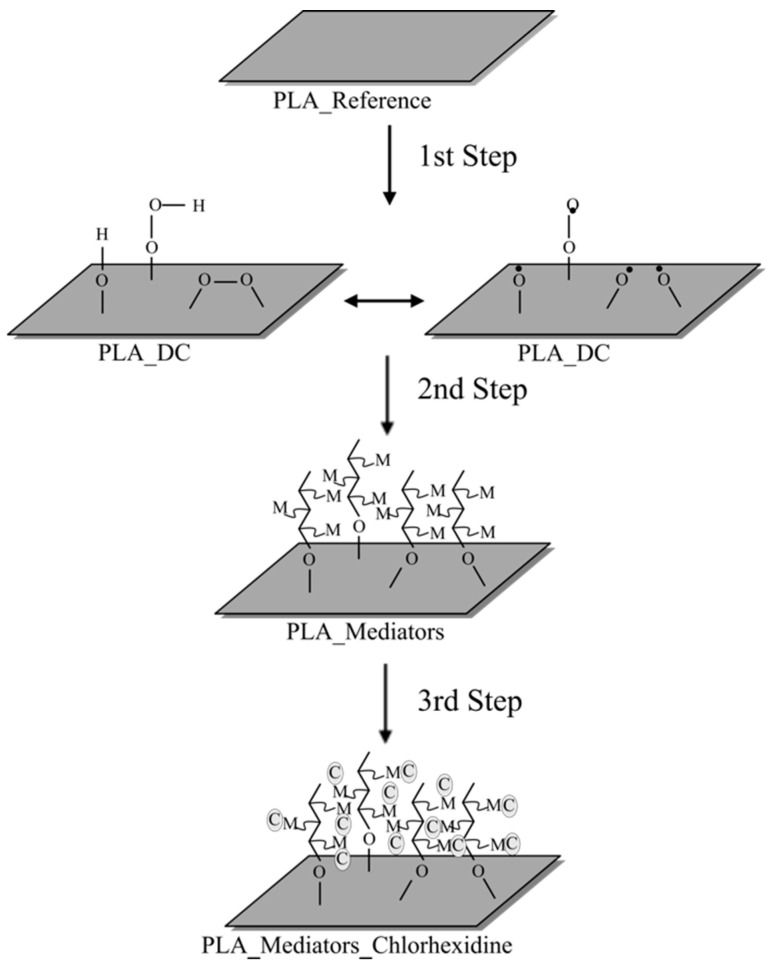
Multistep physicochemical process of the immobilization: first step is plasma treatment to functionalize the PLA surface; second step is the activation of the mediators of EDAC and NHs; the third and final step is the immobilization of CHx onto the activated groups of EDAC and NHs to form amide bonds.

Prior to antibacterial chlorhexidine immobilization, functionalized PLA surfaces were activated by EDAC and NHs mediators through activation of carboxylic acid groups and hydroxyl groups for subsequent covalent bonding with amine groups of CHx. Immediately after the plasma treatment, samples were placed into solution vials containing 0.1% (*w*/*v*) EDAC and NHs, separately, for 24 h on a shaker at room temperature, then gently washed three times with distilled water and dried at room temperature of the ambient air overnight. EDAC and NHs activated samples will be hereafter referred to as PLA_DC_EDAC and PLA_DC_NHs, respectively. As the last step of the CHx immobilization process, samples were placed into the solution vials containing chlorhexidine dissolved in 70% (*v*/*v*) isopropanol for 24 h on a shaker. Finally, all samples were gently washed with distilled water three times and dried at room temperature overnight; hereafter referred to as PLA_DC_EDAC_CHx and PLA_DC_NHs_CHx. The schematic representation of the multistep physicochemical immobilization process is depicted in Figure 2.

### 2.4. Surface Wettability Essay

Evaluation of the surface wettability of the samples was carried out by the sessile drop method via a SEE System (Advex Instruments, Brno-Komín, Czech Republic) equipped with a charge coupled device (CCD) camera. Distilled water droplets of 5 μL were separately placed onto each sample at 22.4 °C and 59% relative humidity for 30 s to reach stable equilibrium. The mean value of the water contact angle (Q_w_) was calculated from ten water droplets for each sample.

### 2.5. Surface Morphology Characterization by Scanning Electron Microscope

The surface morphology was investigated by a NANOSEM 450 (FEI, Morristown, NJ, USA) scanning electron microscope (SEM) to reveal immobilized species of the chlorhexidine onto the PLA surface. SEM equipped with a low vacuum detector operated at 5 kV under 90 Pa pressure in a water vapor environment.

### 2.6. Surface Chemical Analysis by X-ray Photoelectron Spectroscopy

The observation of the changes in chemical composition was carried out by a TFA X-ray photoelectron spectroscopy (Physical Electronics, Chanhassen, MN , USA) with MultiPak v7.3.1 software to fit the collected spectra. The samples were exposed to monochromatic Al K_α1,2_ radiation at 1486.6 eV for a 400 μm spot area under the chamber pressure of 6 × 10^−8^ Pa. The emitted photoelectrons were detected with a hemispherical analyzer placed at an angle of 45° to correlate with the normal plane of the samples.

### 2.7. Antibacterial Test

The antibacterial activity was performed according to ISO 22,196 with modifications, using bacterial strains of *Staphylococcus aureus* (CCM 4516) as Gram-positive and *Escherichia coli* (CCM 4517) as Gram-negative representatives. Samples were prepared with the dimensions of 25 × 25 mm and disinfected by rinsing with 70% ethanol immediately before testing. Bacterial suspensions were prepared in 1/500 nutrient broth (HiMedia Laboratories, Mumbai, India) for *E. coli* of 1.4 × 10^7^ CFU mL^−1^ and *S. aureus* of 4.6×10^6^ CFU mL^−1^. Each bacterial suspension was dispensed on the sample surfaces with a volume of 100 µL, covered with polypropylene foil (20 × 20 mm), and subsequently cultivated at 35 °C and 100% relative humidity for 24 h. After cultivation, polypropylene foils were removed and samples were gently washed by SCDLP broth (HiMedia Laboratories, India). Finally, the viable bacteria count was determined using the pour plate culture method (PCA, HiMedia Laboratories, Mumbai, India).

### 2.8. Cytocompatibility Assay

In vitro cytocompatibility was studied using primary mouse embryonic fibroblast cells (NIH/3T3, ATCC^®^ CRL-1658^TM^, USA). The samples were prepared with dimensions of 10 × 10 mm foil and sterilized under UV-radiation (wavelength of 253.7 nm) for 30 min. As a culture medium, the ATCC-formulated Dulbecco’s modified Eagle’s medium (BioSera, France) containing 10% calf serum (BioSera, France) and 100 U mL^−1^ penicillin/streptomycin (BioSera, France). The cells were seeded onto the samples in a concentration of 2 × 10^4^ cells per mm^2^ and placed in an incubator for 24 h at 37 °C.

After the incubation period, the cell viability was determined using the 3-(4,5-Dimethylthiazol-2-yl)-2,5-diphenyltetrazolium bromide (MTT) assay (Duchefa Biochemie, Amsterdam, the Netherlands). First, the cells were washed with PBS (BioSera, France) and a fresh medium containing MTT in the concentration of 0.5 mg per mL was added. After 4 h, formed formazan crystals were dissolved in DMSO and the absorbance was measured by an Infinite M200 Pro NanoQuant absorbance reader (Tecan, Switzerland) at 570 nm and the reference wavelength was adjusted to 690 nm. The results are presented as a reduction of cell viability in percentage when compared to cells cultivated on pure PLA. All tests were performed three times.

### 2.9. Statistical Analysis

All analysis performed in the manuscript were in triplicate, and one-way analysis of ANOVA was performed. The GraphPad Prism software (Version 6.04, San Diego, CA, USA) was used and *p* < 0.05 was considered as statistically significant.

## 3. Results and Discussion

### 3.1. Surface Wettability Results

**Figure 3:**
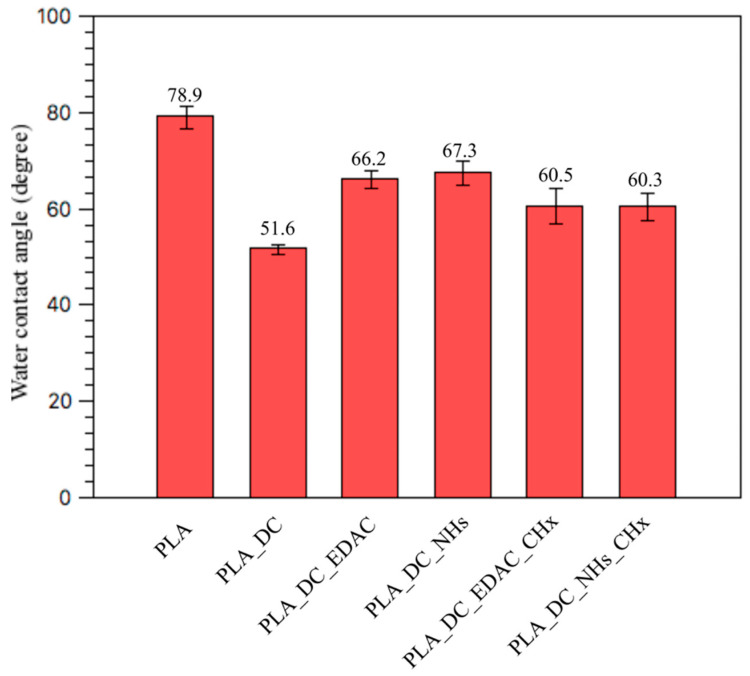
Water contact angle values of the samples.

The water contact angle test was performed by the sessile drop method to reveal the surface wettability of the sample surfaces, which is related to its surface energy, as a result of the interactions between the charges of the water molecules and polar contents of the surface. The water contact angle value of 78.9° ± 2.3 was measured for the reference PLA sample, as the highest contact angle value, which refers to the most hydrophilic surface among all samples (Table 1). Thus, the pure PLA surface had the lowest surface energy and therefore is not convenient for further chemical bonding. The water contact angle value drastically dropped to 51.6° ± 1.0 for the PLA_DC sample due to the incorporation of oxidative hydrophilic functional groups by applying direct current plasma and an increased surface area by plasma etching. Therefore, appropriate surface condition for further chemical immobilization was obtained after plasma treatment by increasing the hydrophilicity and incorporating the oxidative functional groups. As listed in Figure 3, EDAC and NHs activated samples of PLA_DC_EDAC and PLA_DC_NHs displayed similar increased water contact angle values of 66.2° ± 1.8 and 67.3° ± 2.4, respectively, compared to the PLA_DC sample. This indicates the successful bonding of the mediators of EDAC and NHs, and increased water contact angle values. Similarly, CHx immobilized samples of PLA_DC_EDAC_CHx and PLA_DC_NHs_CHx showed the same hydrophilic nature with 60.5° ± 3.7 and 60.3° ± 2.8 contact angle values respectively, as expected, the same molecule was immobilized onto the mediators, which had a higher hydrophilic nature than that of EDAC and NHs. Change in the water contact angle is an indicator of the changes in surface conditions and immobilization of the mentioned chemicals, as was demonstrated by XPS and SEM analysis.

### 3.2. Surface Chemical Analysis Results

The surface chemical composition of the samples was investigated by x-ray photoelectron spectroscopy (XPS) and the results are listed in Table 1. The effect of plasma treatment on the PLA surface can be seen through the changes in the carbon and oxygen levels. The carbon level decreased from 73.7 to 69.8% while the oxygen level increased from 25.3 to 30.2% due to incorporated oxidative groups by plasma. The decrease in oxygen level was detected for the following mediators and CHx immobilized samples as evidence of successfully immobilized chemicals onto the plasma applied PLA surface. Correspondingly, nitrogen and chlorine levels were detected referring to the presence of immobilized chemicals on the surface. The presence of chlorine signifies CHx immobilization and the maximum amount of chlorine was detected for sample PLA_DC_EDAC_CHx, which refers to the highest concentration of immobilized CHx achieved after EDAC activated surface by 3.8%. According to XPS data, the level of chlorine detected was 1% for the sample of PLA_DC_NHs_CHx, which was lower than that of PLA_DC_EDAC_CHx. Therefore, using the EDAC as a mediator led to a higher amount of CHx bonding on the surface, compared to NHs, as was also proven by SEM analysis, due to the different stability and reactivity of the intermediate. However, the level of bonded CHx is not directly related to its antibacterial effect, but the release of CHx from the surface also needs to be considered, which is discussed in the following sections.

### 3.3. Surface Morphology Results by Scanning Electron Microscopy (SEM)

**Figure 4:**
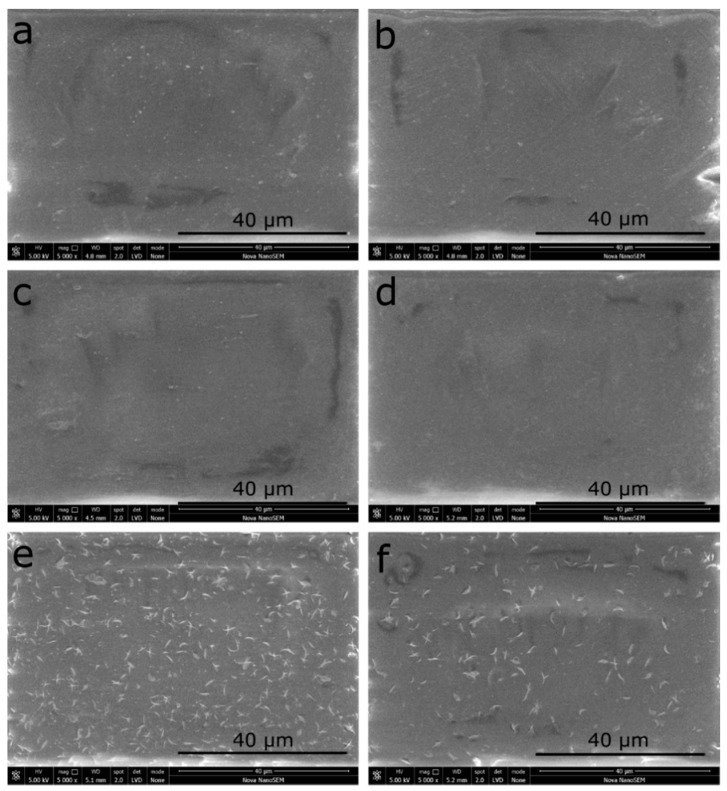
Surface morphology of the samples taken by scanning electron microscopy. (**a**) PLA, (**b**) PLA_DC, (**c**) PLA_DC_EDAC, (**d**) PLA_DC_NHs, (**e**) PLA_DC_EDAC_CHx, and (**f**) PLA_DC_NHs_CHx.

Surface morphological features of the samples were investigated using a scanning electron microscope (SEM) to reveal immobilized chlorhexidine particles, and the results are shown in Figure 4. As can be seen in Figure 4A,B, DC plasma treatment onto the PLA surface did not cause a major effect on its morphology, as obtained by SEM. Likewise, bonding of the mediators of EDAC and NHs onto the PLA_DC sample did not change the morphology of the surface, and particles of the mediators were not visible in the SEM images of Figure 4C,D. However, as seen in Figure 4E,F, CHx was immobilized onto the mediators of EDAC and NHs. In both cases, their particle size, distribution, and morphologies were similar. According to Figure 4E, it seems that CHx with the mediator of EDAC is present in a higher amount compared to the NHs counterpart (Figure 4F). This was also confirmed by the XPS results, showing that the chlorine content was 3.8 times higher for PLA_DC_EDAC_CHx, compared to PLA_DC_NHs_CHx. Nevertheless, the concentration of immobilized CHx was not the only parameter to perform better antibacterial performance, but also how the CHx molecules were displaced on the surface to interact with bacteria.

### 3.4. Antibacterial Activity Results

**Figure 5:**
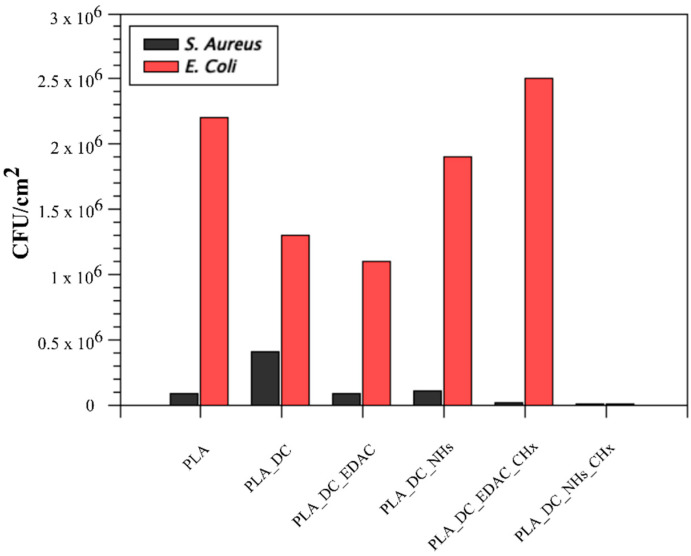
Antibacterial activity graph of the samples with the highest antibacterial performance of PLA_DC_NHs_CHx.

Antibacterial activity of the prepared PLA scaffolds was tested against *Staphylococcus aureus* and *Escherichia coli* as a representative of Gram-positive and Gram-negative bacterial strains and the results are listed in Table 2. The reference PLA scaffold (PLA) did not display any antibacterial activity as the raw PLA itself has no antibacterial nature, however, *E. coli* contamination observed was 25 times higher compared to *S. aureus*, as depicted in Figure 5. Plasma treatment decreased the *E. coli* contamination by half but increased the *S. aureus* contamination by five times compared to the untreated counterpart. EDAC and NHs activated samples displayed a similar lack of antibacterial performance, indicating their non-antibacterial effect, as seen in Figure 5.

The antibacterial agent of CHx immobilized sample of PLA_DC_EDAC_CHx displayed a slightly antibacterial effect against *S. aureus*, but it was not effective against *E. coli*. However, PLA_DC_NHs_CHx samples were effective against both Gram-positive *S. aureus* and Gram-negative *E. coli* strains. The immobilization of CHx was confirmed by SEM micrographs as CHx particles were observed for both CHx samples and with the presence of Cl2p% by XPS results. As seen in Figure 4 and Table 1, the amount of immobilized CHx found was higher for the sample PLA_DC_EDAC_CHx but its antibacterial activity was lower than that of PLA_DC_NHs_CHx. This may be due to the different availability of CHx to interact with bacterial cells [34,35]. The CHx was found to be less effective against *E. coli* and highly effective against *S. aureus* in our study, as was also demonstrated by Castillo et al. [36].

### 3.5. Cytocompatibility Results

Besides antibacterial activity, the biocompatibility of the antibacterial-containing biomaterials is crucial since most of the antibacterial agents are toxic to cells, therefore their usage needs to be moderated to do not induce any adverse effect on surrounding tissue. The most common and relevant marker of biocompatibility is to determine the cytotoxicity of the material, which can be studied either by extracts or by direct contact with cells. In the case of surface modification, direct contact with cells is a more relevant method as the mass of coated film is small compared to the bulk material, and the amount of leaching substance is low. Thus, the cytocompatibility was determined using the direct contact of cells with the surface of materials.

**Figure 6:**
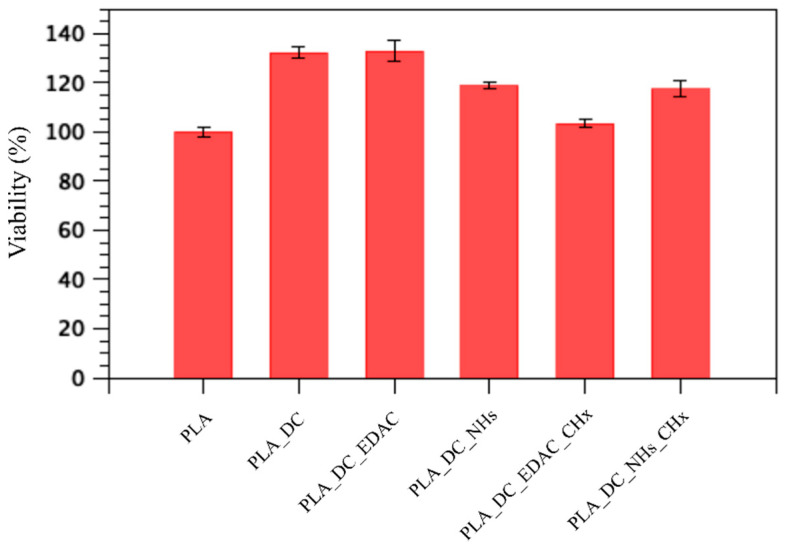
Cytotoxicity results of the samples, compared to reference PLA sample.

As seen in Figure 6 and Table 2, all types of modifications allowed for the adhesion and growth of cells. It is remarkable that the modification of the PLA surface increased the cytocompatibility of PLA as the number of cells presented on its surface was higher. Samples PLA_DC and PLA_DC_EDAC exhibited the highest cytocompatibility. This result was expected as the plasma treatment increased the hydrophilic character of PLA and the wettability of the surface affects cell behavior, especially the cell attachment [37,38,39,40,41]. However, without further immobilization of chlorhexidine, these samples did not exhibit any antibacterial effect. Nevertheless, cell viability decreased by 30% after the immobilization of chlorhexidine to the PLA_DC_EDAC sample. On the other hand, there were no differences in cell viability in the PLA_DC_NHs sample before and after the immobilization of chlorhexidine. Regarding both samples with chlorhexidine, PLA_DC_EDAC_CHx and PLA_DC_NHs_CHx showed better viability. Compared to the PLA, the viability reached 117.6%, whilst PLA_DC_EDAC_CHx had a lower viability by 14%. This can be explained by the fact that chlorhexidine had a stronger bond to the PLA_DC_EDAC sample compared to the PLA_DC_NHs sample, which was proven through the XPS results as well as by the SEM results (Table 1 and Figure 4). In any case, the PLA_DC_NHs_CHx sample shows promising conditions for cell growth together with high antibacterial activity.

## 4. Conclusions

Antibacterial agent, chlorhexidine was successfully immobilized onto the hydrophobic polylactic acid surface after plasma treatment followed by EDAC and NHs activation. The antibacterial effect of functionalized surfaces was tested against *S. aureus* and *E. coli* strains and the cytocompatibility assay was studied using fibroblast cells. It was demonstrated by XPS and SEM analysis that a higher amount of CHx was immobilized onto the EDAC activated surface (PLA_DC_EDAC_CHx) compared to the NHs activated counterpart (PLA_DC_NHs_CHx). However, the antibacterial effect of the PLA_DC_NHs_CHx sample demonstrated a higher effect, especially against *E. coli*. Furthermore, cell adhesion and growth were higher on the PLA_DC_NHs_CHx sample compared to PLA_DC_EDAC_CHx. Thus, the use of NHs, rather than EDAC, was found to be more effective for CHx immobilization onto the PLA surface to achieve higher antibacterial activity and cytocompatibility.

## Figures and Tables

**Table 1:** X-ray photoelectron spectroscopy with observed carbon (C1s%), oxygen (O1s), nitrogen (N1s%), chlorine (Cl2p%) levels and water contact angle (Q_w_ °) results. * Standard deviation up to 3% (n = 3).

Samples	C1s% *	O1s% *	N1s% *	Cl2p% *	Q_w_ °
PLA	73.7	25.3	1	0	78.9 ± 2.3
PLA_DC	69.8	30.2	0	0	51.6 ± 1.0
PLA_DC_EDAC	68.9	29.6	1.4	0.2	66.2 ± 1.8
PLA_DC_NHs	66.8	32.6	0.5	0.1	67.3 ± 2.4
PLA_DC_EDAC_CHx	69	16.4	10.8	3.8	60.5 ± 3.7
PLA_DC_NHs_CHx	66.3	28.4	4.3	1	60.3 ± 2.8

**Table 2:** Antibacterial and cytocompatibility test results using colony-forming units per cm^2^ and cell viability.

Samples	*S. aureus,* N (CFU/cm^2^)	*E. coli*, N (CFU/cm^2^)	Cell Viability (%)
PLA	8.8 × 10^4^	2.2 × 10^6^	100 ± 1.7
PLA_DC	4.1 × 10^5^	1.3 × 10^6^	132.2 ± 2.1
PLA_DC_EDAC	8.3 × 10^4^	1.1 × 10^6^	132.7 ± 4.2
PLA_DC_NHs	1.1 × 10^5^	1.9 × 10^6^	118.6 ± 1.2
PLA_DC_EDAC_CHx	1.2 × 10^4^	2.5 × 10^6^	103.4 ± 1.9
PLA_DC_NHs_CHx	6.2 × 10^3^	6.0 × 10^1^	117.6 ± 3.1

## References

[1] Carrasco F., Pages P., Gamez-Perez J., Santana O.O., Maspoch M.L. (2010). Processing of poly(lactic acid): Characterization of chemical structure, thermal stability and mechanical properties. Polym. Degrad. Stab..

[2] Garlotta D. (2001). A literature review of poly(lactic acid). J. Polym. Environ..

[3] Lehocky M., Stahel P., Koutny M., Cech J., Institoris J., Mracek A. (2009). Adhesion of Rhodococcus sp. S3E2 and Rhodococcus sp. S3E3 to plasma prepared Teflon-like and organosilicon surfaces. J. Mater. Process. Technol..

[4] Adamczyk Z., Szyk-Warszynska L., Zembala M., Lehocky M. (2004). In situ studies of particle deposition on non-transparent substrates. Colloids. Surf. A Physicochem. Eng. Asp..

[5] Asadinezhad A., Lehocky M., Saha P., Mozetic M. (2012). Recent Progress in Surface Modification of Polyvinyl Chloride. Materials.

[6] Bilek F., Sulovska K., Lehocky M., Saha P., Humpolicek P., Mozetic M., Junkar I. (2013). Preparation of active antibacterial LDPE surface through multistep physicochemical approach II: Graft type effect on antibacterial properties. Colloids Surf. B Biointerfaces.

[7] Kale R.D., Gorade V.G., Madye N., Chaudhary B., Bangde P.S., Dandekar P.P. (2018). Preparation and characterization of biocomposite packaging film from poly(lactic acid) and acylated microcrystalline cellulose using rice bran oil. Int. J. Biol. Macromol..

[8] Lehocky M., Amaral P.F.F., Coelho M.A.Z., Stahel P., Barros-Timmons A.M., Coutinho J.A.P. (2006). Attachment/detachment of Saccharomyces cerevisiae on plasma deposited organosilicon thin films. Czechoslov. J. Phys..

[9] Bilek F., Krizova T., Lehocky M. (2011). Preparation of active antibacterial LDPE surface through multistep physicochemical approach: I. Allylamine grafting, attachment of antibacterial agent and antibacterial activity assessment. Colloids Surf. B Biointerfaces.

[10] Asadinezhad A., Novak I., Lehocky M., Sedlarik V., Vesel A., Junkar I., Saha P., Chodak I. (2010). An in vitro bacterial adhesion assessment of surface-modified medical-grade PVC. Colloids Surf. B Biointerfaces.

[11] Junkar I., Cvelbar U., Lehocky M. (2011). Plasma treatment of biomedical materials. Mater. Tehnol..

[12] Cao H., Liu X. (2010). Silver nanoparticles-modified films versus biomedical device-associated infections. Wiley Interdiscip. Rev. Nanomed. Nanobiotechnol..

[13] Lehocky M., Amaral P.F.F., Stahel P., Coelho M.A.Z., Barros-Timmons A.M., Coutinho J.A.P. (2008). Deposition of Yarrowia lipolytica on plasma prepared teflonlike thin films. Surf. Eng..

[14] Hughes C., Ferguson J. (2017). Phenotypic chlorhexidine and triclosan susceptibility in clinical Staphylococcus aureus isolates in Australia. Pathology.

[15] Greenhalgh R., Dempsey-Hibbert N.C., Whitehead K.A. (2019). Antimicrobial strategies to reduce polymer biomaterial infections and their economic implications and considerations. Int. Biodeterior. Biodegrad..

[16] Lobato-Aguilar H., Uribe-Calderon J.A., Herrera-Kao W., Duarte-Aranda S., Baas-Lopez J.M., Escobar-Morales B., Cauich-Rodriguez J.V., Cervantes-Uc J.M. (2018). Synthesis, characterization and chlorhexidine release from either montmorillonite or palygorskite modified organoclays for antibacterial applications. J. Drug Deliv. Sci. Technol..

[17] Scheibler E., da-Silva R.M., Leite C.E., Campos M.M., Figueiredo M.A., Salum F.G., Cherubini K. (2018). Stability and efficacy of combined nystatin and chlorhexidine against suspensions and biofilms of Candida albicans. Arch. Oral. Biol..

[18] Kao H.F., Chen I.C., Hsu C., Chang S.Y., Chien S.F., Chen Y.C., Hu F.C., Yang J.C.H., Cheng A.L., Yeh K.H. (2014). Chlorhexidine for the prevention of bloodstream infection associated with totally implantable venous ports in patients with solid cancers. Support. Care Cancer.

[19] Keerthisinghe T.P., Nguyen L.N., Kwon E.E., Oh S. (2019). Antiseptic chlorhexidine in activated sludge: Biosorption, antimicrobial susceptibility, and alteration of community structure. J. Environ. Manag..

[20] Zhang Y., Zhao Y., Xu C., Zhang X., Li J., Dong G., Cao J., Zhou T. (2019). Chlorhexidine exposure of clinical Klebsiella pneumoniae strains leads to acquired resistance to this disinfectant and to colistin. Int. J. Antimicrob. Agents.

[21] Bonez P.C., Alves C.F.D.S., Dalmolin T.V., Agertt V.A., Mizdal C.R., Flores V.D.C., Marques J.B., Santos R.C.V., Campos M.M.A.D. (2013). Chlorhexidine activity against bacterial biofilms. Am. J. Infect. Control..

[22] Popelka A., Novak I., Lehocky M., Chodak I., Sedliacik J., Gajtanska M., Sedliacikova M., Vesel A., Junkar I., Kleinova A. (2012). Anti-bacterial treatment of polyethylene by cold plasma for medical purposes. Molecules.

[23] Vesel A., Mozetic M. (2012). Surface modification and ageing of PMMA polymer by oxygen plasma treatment. Vacuum.

[24] Lehocky M., Lapcik L., Neves M.C., Trindade T., Szyk-Warszynska L., Warszynski P., Hui D. (2003). Deposition/Detachment of Particles on Plasma Treated Polymer Surfaces. Mater. Sci. Forum.

[25] Lehocky M., Lapcik L., Dlabaja R., Rachunek L., Stoch J. (2004). Influence of artificially accelerated ageing on the adhesive joint of plasma treated polymer materials. Czech. J. Phys..

[26] Patel D., Wu J., Chan P., Upreti S., Turcotte G., Ye T. (2012). Surface modification of low density polyethylene films by homogeneous catalytic ozonation. Chem. Eng. Res. Des..

[27] Ding Q., Xu X., Yue Y., Mei C., Huang C., Jiang S., Wu Q., Han J. (2018). Nanocellulose-Mediated Electroconductive Self-Healing Hydrogels with High Strength, Plasticity, Viscoelasticity, Stretchability, and Biocompatibility toward Multifunctional Applications. ACS Appl. Mater. Interfaces.

[28] Lopez-Garcia J., Bilek F., Lehocky M., Junkar I., Mozetic M., Sowe M. (2013). Enhanced printability of polyethylene through air plasma treatment. Vacuum.

[29] Mozetic M. (2020). Plasma-Stimulated Super-Hydrophilic Surface Finish of Polymers. Polymers.

[30] Niemczyk-Soczynska B., Arkadiusz Gradys A., Sajkiewicz P. (2020). Hydrophilic Surface Functionalization of Electrospun Nanofibrous Scaffolds in Tissue Engineering. Polymers.

[31] Wieland F., Bruch R., Bergmann M., Partel S., Urban G.A., Dincer C. (2020). Enhanced Protein Immobilization on Polymers—A Plasma Surface Activation Study. Polymers.

[32] Slepicka P., Kasalkova N.S., Stranska E., Bacakova L., Svorcik V. (2013). Surface characterization of plasma treated polymers for applications as biocompatible carriers. Express Polym. Lett..

[33] Vidaurre E.F.C., Achete C.A., Gallo F., Garcia D., Simao R., Habert A.C. (2002). Surface Modification of Polymeric Materials by Plasma Treatment. Mater. Res..

[34] Asadinezhad A., Novak I., Lehocky M., Sedlarik V., Vesel A., Junkar I., Saha P., Chodak I. (2010). A physicochemical approach to render antibacterial surfaces on plasma-treated medical-grade PVC: Irgasan coating. Plasma Process. Polym..

[35] Asadinezhad A., Novak I., Lehocky M., Bilek F., Vesel A., Junkar I., Saha P., Popelka A. (2010). Polysaccharides Coatings on Medical-Grade PVC: A Probe into Surface Characteristics and the Extent of Bacterial Adhesion. Molecules.

[36] Castillo J.A., Clapes P., Infante M.R., Comas J., Manresa A. (2006). Comparative study of the antimicrobial activity of bis(Nα-caproyl-L-arginine)-1,3-propanediamine dihydrochloride and chlorhexidine dihydrochloride against Staphylococcus aureus and Escherichia coli. J. Antimicrob. Chemother..

[37] Ozaltin K., Lehocky M., Kucekova Z., Humpolicek P., Saha P. (2017). A novel multistep method for chondroitin sulphate immobilization and its interaction with fibroblast cells. Mater. Sci. Eng. C.

[38] Esmail A., Pereira J.R., Zoio P., Silvestre S., Menda U.D., Sevrin C., Grandfils C., Fortunato E., Reis M.A.M., Henriques C. (2021). Oxygen Plasma Treated-Electrospun Polyhydroxyalkanoate Scaffolds for Hydrophilicity Improvement and Cell Adhesion. Polymers.

[39] Karakurt I., Ozaltin K., Vesela D., Lehocky M., Humpolicek P., Mozetic M. (2019). Antibacterial Activity and Cytotoxicity ofImmobilized Glucosamine/Chondroitin Sulfate on Polylactic Acid Films. Polymers.

[40] Ozaltin K., Vargun E., Martino A.D., Capakova Z., Lehocky M., Humpolicek P., Kazantseva N., Saha P. (2020). Cell response to PLA scaffolds functionalized with various seaweedpolysaccharides. Int. J. Polym. Mater. Polym. Biomater..

[41] Bernal-Ballen A., Lopez-Garcia J.A., Ozaltin K. (2020). (PVA/Chitosan/Fucoidan)-Ampicillin: A BioartificialPolymeric Material with Combined Properties in CellRegeneration and Potential Antibacterial Features. Polymers.

